# Impact of hepatitis C virus co-infection on HIV patients before and after highly active antiretroviral therapy: an immunological and clinical chemistry observation, Addis Ababa, Ethiopia

**DOI:** 10.1186/1471-2172-14-23

**Published:** 2013-05-17

**Authors:** Solomon Taye, Mekuria Lakew

**Affiliations:** 1 Madawalabu University, College of Medicine and Health Sciences, P.O. Box 302, Bale Goba, Ethiopia; 2 Faculty of Life Sciences, Department of Biomedical Sciences, Addis Ababa University, P. O. Box 1176, Addis Ababa, Ethiopia

**Keywords:** Immunological, HCV/HIV co-infection, Pre-ART, HAART, CD4^+^, CD8^+^, GOT, GPT, Alkaline phosphatase

## Abstract

**Background:**

Hepatitis C virus (HCV) is an RNA virus which has been known to cause acute and chronic necro-inflammatory disease of the liver. It is the leading cause of end-stage liver disease and hepatocellular carcinoma. HIV is known to have a negative impact on the natural disease outcome and immune response of HCV infection, whereas the reverse remains unclear. We evaluated the impact of HCV co-infection on recovery of CD4^+^ and CD8^+^ T-cells and liver enzyme levels before and after initiation of highly active antiretroviral therapy (HAART) in HIV/HCV co-infected patients.

**Methods:**

A hospital-based, observational, prospective cohort study design was used for this study. Pre-antiretroviral treatment (Pre-ART) and under HAART HIV mono-infected and HCV/HIV co-infected individuals who are under regular follow-up were recruited for this study. 387 blood samples were collected from volunteer, known HIV positive Ethiopian patients and screened for HCV. Twenty five HCV/HIV co-infected patients were prospectively followed for four years. CD4^+^ and CD8^+^ T-cells and liver enzyme levels were determined annually for each of the participant.

**Results:**

The prevalence of HCV/HIV co-infection in this study was 6.5%. Both HCV/HIV co-infected and HIV mono-infected under HAART groups showed CD4^+^ recovery (343 Vs 426; *P < 0.004,* OR = 4.97, 95% CI = 2.41 to 10.27) respectively; but, the recovery rate was higher in mono-infected (80 Vs 426) than co-infected group (148 Vs 343). The recovery and/or decline pattern of CD8^+^ T-cells was the same with that of CD4^+^. In 75% of co-infected groups, the mean alanine aminotransferase (ALT) and aspartate aminotransferase (AST) levels were above the upper limit of normal reference range. Analyses restricted to individuals who initiated HAART and pre-ART showed similar results.

**Conclusion:**

We found that CD4^+^ T-cell recovery was negatively affected by the presence of ongoing HCV replication in under HAART co-infected individuals and fast decline of CD4^+^ T-cells in pre-ART patients. It was also associated with increased ALT and AST enzyme levels in both HAART initiated and treatment naïve co-infected patients.

## Background

Hepatitis C virus (HCV) is a RNA virus which has been known to cause acute and chronic necroinflamatory disease of the liver. It infects more than 170 million people worldwide. In Western countries, HCV is the leading cause of end-stage liver disease and hepatocellular carcinoma, as well as the main indication for liver transplantation [1,2]. Because of shared routes of transmission, co-infection with HCV and HIV is quite common. In the era of HAART, HCV-related liver disease has emerged as a significant cause of morbidity and mortality due to the increased risk for hepatotoxicity of HAART and likelihood of onset of an AIDS-defining illness [3].

Both innate and Cell-mediated immune responses are crucial in the early control of viral infections. Although the role of T-cell immunity during acute and chronic HCV infection and its relationship with HCV replication remains controversial, CD4^+^ T-cell responses particularly to non-structural HCV proteins, specific CD8^+^ T cell cytolytic action, and high level local gamma-interferon production are believed to be important [4]. It has been suggested that the deficiency of cell-mediated immune response against HIV infection actually favors the chronic development of acute HCV infection and also the progression of chronic hepatitis to cirrhosis [5]. HCV/ HIV co-infection often associated with elevated liver biochemical enzymes such as alanine aminotransferase, aspartate aminotransferase and alkaline phosphatase [6].

Although the co-infection of HIV with HCV has been recognized worldwide in individuals exposed to blood borne diseases, limited data are available on the extent of co-infection, effect of these viruses on the immune system and liver in developing countries. Ethiopia belongs to the group of countries which are highly endemic for viral hepatitis. Few studies have been done on HIV/HCV co-infection prevalence in Ethiopia but the knowledge about the interrelationship between these viruses and their effect on the immune system remains unclear [7].

Therefore, the aim of this study was to estimate the prevalence of HCV sero-positivity in a cohort of people living with HIV in Addis Ababa and to investigate its effect on the recovery of CD4^+^ and CD8^+^ T-lymphocytes and liver enzymes in the era of HAART and before HAART in Ethiopia.

## Methods

### Study design, population and sampling technique

A hospital-based, observational, prospective cohort study design was used for this study. The study population was comprised of HIV patients on follow-up and VCT attendants at Yekatit-12 and Zenbaba General Hospitals in September 2006, Addis Ababa, Ethiopia. From September 1–30, 2006, a total of 387 HIV positive patients were screened for HCV and HBV. Hence, a convenient, non-probability sampling technique was employed and no scientific method were used to calculate the sample size, instead, we screened all eligible patients who visited the two hospitals during the month September 2006 and we followed volunteer 25 HCV/HIV co-infected patients. The control groups (HCV negatives) were also screened for HBV infection.

All those 25 HCV/HIV co-infected and 25 HIV mono-infected patients were enrolled in this hospital-based observational follow-up study. All selected patients were paired on the basis of world health organization (WHO) clinical disease stages (All were WHO stage II patients). The study groups were prospectively followed for four years from Sep 2006 to Nov 2010 in order to determine the impact of HCV/HIV co-infection on immunological and liver enzyme levels of HIV patients.

### Patient grouping

Selected study cohort participants were arranged in to four groups based on their HCV and HAART (status: I) 16 under HAART patients only with HIV infection (Group 1), II) 16 HCV/HIV co-infected patients receiving HAART (Group 2), III) 9 HIV positive pre-ART patients without HCV infection (Group 3) and IV) 9 HIV/HCV co-infected pre-ART patients (Group 4). Both co-infected and HIV mono-infected under HAART patients has been taking the same combination therapy (Zidovudine (ZDV) + Lamivudine (3TC) + Efavirenz) and there were no considerable difference on the duration of treatment initiation between co-infected and HIV mono-infected groups (8 and 7 months) respectively. Annual immunological and clinical chemistry tests were done only for selected patient. The study subjects were also screened for HCV and HBV each year.

#### HCV and HBV screening

Flavicheck-HCV, a commercial fourth generation, rapid, qualitative, two-site sandwich immunoassay test device (Qualpro Diagnostics, 88/89, phase IIC, Verma Industrial Estate, Verna, Goa-403 722, India, 2004) was employed according to the manufacturer’s instructions. It detects total antibodies specific to HCV in serum or plasma. It uses a multi-epitope recombinant peptide antigen that is broadly cross-reactive to all major HCV genotypes. Except the recombinant peptide inserted the principle, procedure and interpretation of HBV are the same with that of HCV.

#### CD4^+^ and CD8^+^ T-cells Enumeration

CD4^+^ and CD8^+^ T-cell counts were enumerated for each patient annually for four consecutive years starting from Sep 2006 through Nov 2010. Both tests were measured by standard 3-color flow cytometry using Fluorescent Activated Cell Sorter (FACscan) machine (Becton Dickinson Biosciences, San Jose, CA 95131–1807, USA). No viral load test was performed for all groups of patients because of scarcity of resources.

#### Measurement of GPT and GOT

Humastar180, chemistry analyzer (Human GmbH.65205 Wiesbaden, Germany) was used to measure the liver enzyme levels (serum GPT, GOT and ALP). Principle of operation is based on the fact that substances of clinical interest selectively absorb or emit energy (light) at different wavelengths.

#### Statistical analysis

Data entry and analysis was done using computer software SPSS version 16. Data was summarized and presented in a descriptive measure such as a table, figures and line graphs. Group comparisons were done using logistic regression, hazard ratio (HR) and odds ratio (OR) to determine the independent effect of the variables by calculating the strength of the association between the infection and risks. Line graph was done to show the trends of CD4^+^ and CD8^+^ T cells counts and liver enzyme levels along the four years follow up. Kaplan-Meier survival analysis done to predict the survival time of HCV/HIV co-infected patients from diagnosis of AIDS to termination of the study. P-value of less than 0.05 was considered statistically significant.

The study protocol was approved by Addis Ababa University, biology department research ethics committee. All participants gave informed and written consent, and HCV-positive cases were contacted with nurses and doctors for further management.

## Result

### Prevalence of HCV/HIV among the study subjects

**Figure 1:**
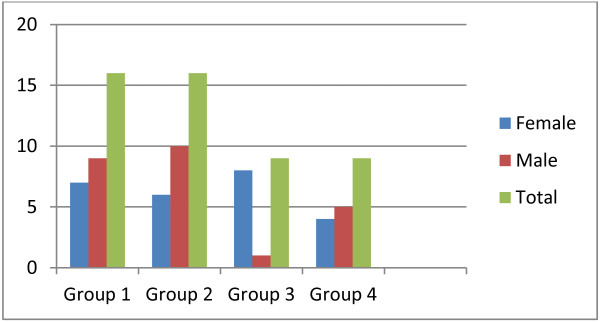
Sex distribution of study subjects in each group at Yekatit-12 and Zenbaba General Hospitals, Addis Ababa (Sep. 2006- Nov. 2010).

**Figure 2:**
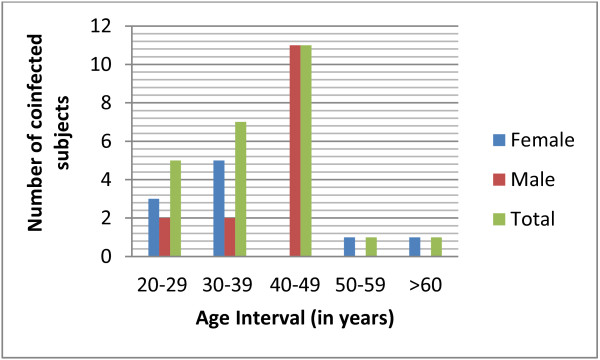
Sex distribution of HCV/HIV co-infected study subjects with age interval at Yekatit-12 and Zenbaba General Hospitals, Addis Ababa (Sep. 2006- Nov. 2010).

The total prevalence of HCV among the 387 HIV patients (182 female and 205 male) who visited the two hospitals in the month September 2006 and were screened for the study was 6.5%. Of these, 206 (53.23%) were under HAART and 181 (46.77%) pre-ART patients. Relatively more HCV infected patients, (7.8%), were from those under HAART and (5%) from pre-ART. The mean age for the patients under investigation was 38.9 years. Even though it was not statistically significant (*p = 0.06*), females were relatively younger (34.5 years) when compared to males (42.6 years). The mean age of patients in the four groups (1–4) was comparable; 41.2, 41.3, 46.2 and 40.2 years respectively, indicating there were no a statistical age differences across the groups (*p = 0.09*). There were also no statistical difference (*p = 0.08*) between the overall mean age and group mean ages of the study subjects (38.9 Vs 41.2, 41.3, 46.2 and 40.2). As shown in Figures 1 and 2, 23 (92%) of the total co-infected patients fall between 20–49, 1 (4%) between 50–59 and 1 (4%) above 60 years of age. In this study, HCV/HIV co-infection was higher in males than females (60% Vs 40%, *p = 0.06*). Furthermore, 11(73.33%) of the co-infected males were over the age of 40 years.

**Figure 3:**
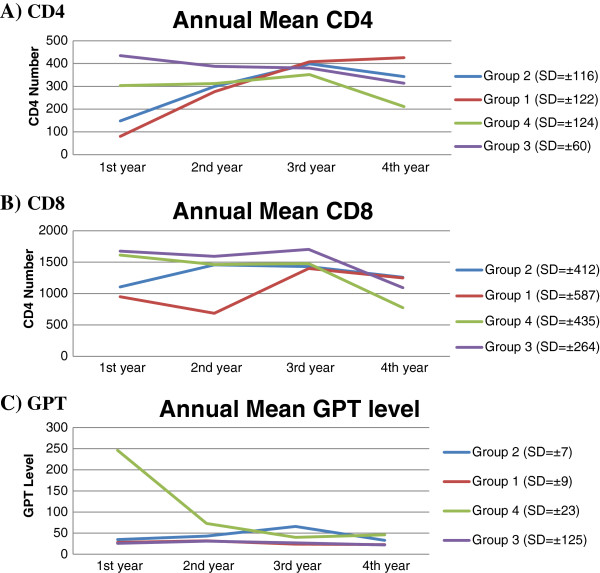
**Line graphs showing trends of CD4**^**+ **^**(A), CD8**^**+ **^**(B) and GPT (C) respectively along the 4 years in all groups of patients.**

Table 1 and Figure 3A-B shows the mean baseline and fourth year CD4^+^ and CD8^+^ cell count of HIV/HCV co-infected and HIV mono-infected under HAART patients. The CD4^+^ and CD8^+^ recovery of co-infected group 2 was impaired by the co-infection of HCV despite the presence of HAART (CD4^+^: 148 Vs 343; *P < 0.003* and CD8^+^: 1104 Vs 1259; *P < 0.004*) at the baseline and the last (fourth year). However, in HIV mono-infected group 1 both variables recovered significantly (CD4^+^: 80 Vs 426; *p < 0.000* and CD8^+^: 950 Vs 1247; *P < 0.003*). Even though both groups showed recovery during the last CD4^+^ count; however, the recovery rate was high in mono-infected group 1 than co-infected group 2 (426 Vs 343; *P < 0.004,* OR = 4.97, 95%CI = 2.41 to 10.27).

**Table 1:** **Mean baseline and fourth year CD4**^**+ **^**and CD8**^**+ **^**cell count of under HAART subjects**

**Cells/μl**	**Year**	**HIV/HCV co-infected (G2) (n = 16) Mean ± SD**	**HIV mono-infected (G1) (n = 16) Mean ± SD**	**p value**
CD4^**+**^	1^st^ year	148 ± 30	80 ± 151	*P < 0.002*
	4^th^ year	343 ± 119	426 ± 113	*P < 0.004*
CD8^**+**^	1^st^ year	1104 ± 420	950 ± 631	*P < 0.003*
	4^th^ year	1259 ± 430	1247 ± 420	*N/S*

(Number of patients in parenthesis), N/S- not significant.

**Key**: G = Group, HAART = highly active antiretroviral therapy, ART = Antiretroviral treatment, μl = microliter, CD = Cluster designation/differentiation, SD = standard deviation.

Table 2 and Figure 3A-B shows the mean baseline and fourth year CD4^+^ and CD8^+^ cell count of HIV/HCV co-infected and HIV mono-infected pre-ART individuals. At the baseline, there was a statistically significant CD4^+^ difference between co-infected and HIV mono-infected groups (303Vs 435; *P < 0.003*) whereas, both groups had a comparable CD8^+^ count (1612 Vs 1676) respectively. The last cell count (fourth year) clearly indicated that HCV co-infection was associated with fast decline of both CD4^+^ and CD8^+^ cells count than HIV mono-infected groups (CD4^+^: 211 Vs 314; *P < 0.004* and CD8^+^: 772 Vs 1092; *P < 0.002*) respectively.

**Table 2:** **Mean baseline and fourth year CD4**^**+ **^**and CD8**^**+ **^**cell count of pre-ART subjects**

**Cells/μl**	**Year**	**HIV/HCV co-infected (G4) (n = 9) Mean ± SD**	**HIV mono-infected (G3) (n = 9) Mean ± SD**	**p value**
CD4^**+**^	1^st^ year	303 ± 73	435 ± 52	*P < 0.003*
	4^th^ year	211 ± 163	314 ± 27	*P < 0.004*
CD8^**+**^	1^st^ year	1612 ± 380	1676 ± 343	*N/S*
	4^th^ year	772 ± 552	1092 ± 177	*P < 0.002*

(Number of patients in parenthesis), N/S- not significant.

**Key**: G = Group, HAART = highly active antiretroviral therapy, ART = Antiretroviral treatment, μl = microliter, CD = Cluster designation/differentiation, SD = standard deviation.

Table 3 shows the mean CD4^+^ and CD8^+^ counts of both co-infected groups of patients at the beginning and fourth year. In the pre-ART group (G4), both CD4^+^ and CD8^+^ count were declined to initiate HAART (303 to 211, *P < 0.004* and 1612 to 772, *P < 0.001*) respectively, whereas, in the HAART group (G2) both cells recovered even though the rate of recovery was impaired by the presence of HCV (CD4^+^: 148 Vs 343; *P < 0.003* and CD8^+^: 1104 Vs 1259; *P < 0.004*) respectively.

**Table 3:** **Mean baseline and fourth year CD4**^**+ **^**and CD8**^**+ **^**cell count of HIV/HCV co-infected under HAART and pre-ART patients**

**Cells/μl**	**Year**	**HIV/HCV co-infected (G2) under HAART (n = 16) Mean ± SD**	**HIV mono-infected (G4) pre-ART (n = 9) Mean ± SD**	**p value**
CD4^**+**^	1^st^ year	148 ± 30	303 ± 73	*P < 0.001*
	4^th^ year	343 ± 119	211 ± 163	*P < 0.004*
CD8^**+**^	1^st^ year	1104 ± 420	1612 ± 380	*P < 0.003*
	4^th^ year	1259 ± 430	772 ± 552	*P < 0.003*

(Number of patients in parenthesis), N/S- not significant.

**Key**: G = Group, HAART = highly active antiretroviral therapy, ART = Antiretroviral treatment, μl = microliter, CD = Cluster designation/differentiation, SD = standard deviation.

**Figure 4:**
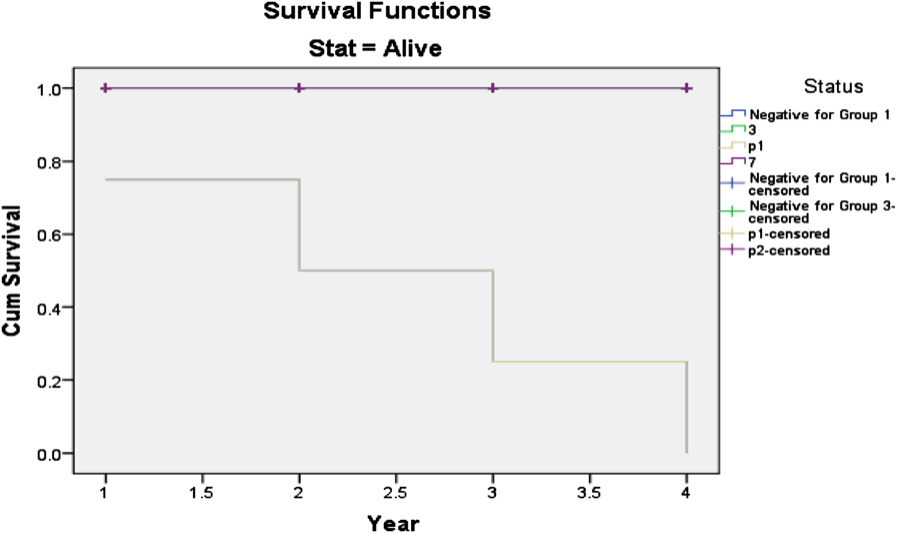
**Survival curve for time from diagnosis of AIDS to termination of the study, stratified by HCV co-infection status, CD4**^**+ **^**cell count and HAART use.**

**Figure 5:**
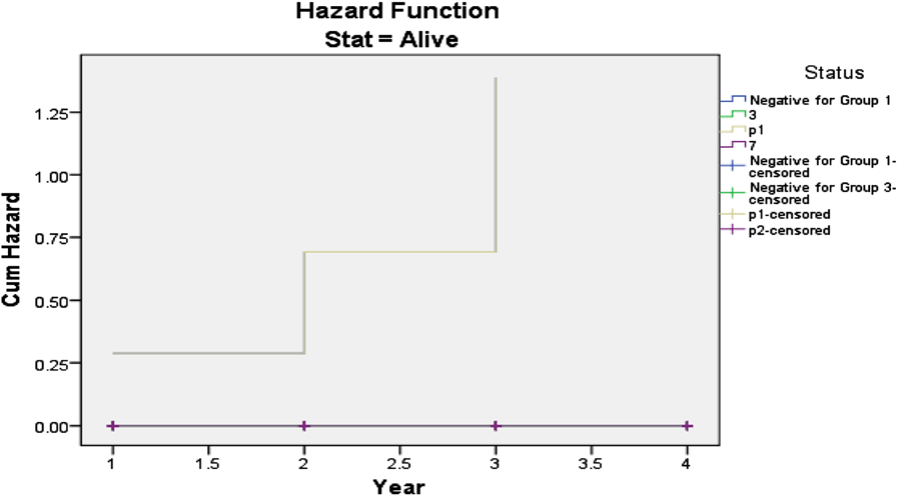
**Hazard curve for HCV/HIV co-infected and HIV mono-infected patients, stratified by HCV co-infection status, CD4**^**+ **^**cell count and HAART use.**

The survival analysis curve shows that, HCV/HIV co-infected patients experienced significantly decreased durations of survival from the time of AIDS diagnosis (hazard ratio ^(^HR), 2.85; 95% CI, 1.16-3.13) (Figure 4). The use of HAART improved survival duration (HR, 0.31; 95% CI, 0.11-0.37). HCV/HIV co-infected patients also experienced shorter durations of survival from the date of diagnosis of HIV infection to AIDS diagnosis than did HIV mono-infected patients (HR, 3.82; 95% CI, 1.32- 4.23) (Figure 5).

Table 4 shows liver enzyme levels of both co-infected under HAART and pre-ART patients at the beginning and fourth year sampling result. It shows a decline to the normal reference values from baseline to the fourth year which indicates the improvement of hepatotoxicity. The pre-ART co-infected group 4 patients improved better (246 IU/L to 46 IU/L) in GPT level than under HAART co-infected group patients (35 IU/L to 33 IU/L). The mean liver enzyme levels of group 1 and group 3 were generally fall within the normal reference range. However, the two co-infected groups had slightly greater than the normal range (Figure 3C and Table 4).

**Table 4:** Mean baseline and fourth year liver enzyme levels of HIV/HCV co-infected under HAART and pre-ART patients

**Enzyme level (IU/l)**	**Year**	**Under HAART co-infected Group (G2) (n = 16), Mean ± SD**	**Pre-ART Co-infected group (G4) (n = 9), Mean ± SD**	**p value**
GPT	1^st^ year	35 ± 5	246 ± 22	P < 0.000
	4^th^ year	33 ± 12	46 ± 10	N/S
GOT	1^st^ year	32 ± 5	207 ± 12	P < 0.000
	4^th^ year	36 ± 14	35 ± 14	N/S
ALP	1^st^ year	243 ± 50	239 ± 93	N/S
	4^th^ year	235 ± 124	207 ± 85	N/S

(Number of patients in parenthesis), N/S- not significant.

**Key**: GPT = Glutamate pyruvate transaminase, GOT = Glutamate oxaloacetate transaminase, ALP = Alkaline phosphatase, IU = International unit, l = liter.

## Discussion

Hepatitis C virus infection is one of the major diseases of mankind and is a serious global public health problem. The precise effect of HCV co-infection on the recovery of immune cells and liver enzymes in HIV patients before and after HAART remains controversial. However, a number of studies have suggested that the presence of HIV infection accelerates the course of HCV-related liver disease in HCV/HIV co-infected patients [5,8]. HIV is known to impair T-helper type 1 immune response which in turn alters the response of immune cells to HCV. This permits greater HCV replication and consequently, greater infection and injury to hepatocytes which leads to more rapid progression to HCV-related liver diseases (fibrosis, cirrhosis and hepatocellular carcinoma) [5].

Given the above interaction of the two viruses and their implications on the proper management of the co-infected cases, the aim of this work was to describe the prevalence of HCV co-infection among HIV patients at Yekatit-12 and Zenbaba General Hospitals and assess the immunological and clinical chemistry results over a four years period. Results show that the prevalence of HCV among the 387 Addis Ababa resident-HIV patients, who visited the two hospitals during one month (September 2006), was 6.5%. This was low in accordance with the previous HIV/HCV co-infection studies in Ethiopia by Workinesh, *et al*. [7] and Addisu *et al.*[2] which was 8.6% and 10.5% respectively. Compared to Spain (33%), U.S.A (30%), France (24.3%) and Morocco (19.8%), the present prevalence was still low [5,9]. The co-infection prevalence in under HAART (7.8%) was more than Pre-ART patients (5%). This might be due to the higher numbers of under HAART (N = 206) than the pre-ART (N = 181) patients. In this study, from the 25 co-infected patients more males were co-infected than females (60% Vs 40%). This might be due to the higher number of males than females in the sample population, hence, does not justify to saying that it is gender influenced.

The distribution of HCV/HIV co-infection, in Figure 2 shows the direct link of age to HCV prevalence. It starts with 20% in age group 20–29 and grows to nearly 45% in age groups 40–49 years suggesting an association with age. A higher prevalence in older age groups could be a reflection due to the chronic nature of the disease, sexual behavior or it may be related to hormone and immunity. Our finding was in agreement with the work of Sugimoto, *et al.*[11] that found HIV/HCV co-infection is higher in males over 40 years of age. This is a serious indicator for over 50-80% of co-infected patients develop chronic infection that gives rise to liver cirrhosis (4-20%) and hepatocellular carcinoma in 1-5% [10].

The observation on immunological parameters over four years showed that an improvement of CD4^+^ and CD8^+^ counts in both HCV positive and negative under HAART patients. However, the CD4^+^ increase in those not co-infected with HCV was much better when compared with those of HCV infected (426 ± 113 Vs 343 ± 119, *P < 0.004*). This suggests that although HAART does improve the immune system of HCV co-infected patients, its efficiency is relatively compromised by HCV interactions (Figure 3 and Tables 1 and 2). Our finding was in agreement with researchers who concluded that even though HAART suppresses HIV and increase CD4^+^ count response, however, it is affected by the presence of HCV co-infection [4]. In addition, HAART adversely affect HCV outcomes by increasing HCV viral load, hepatotoxicity and increased HCV related liver disease progression in HIV/HCV co-infected people [12].

Variety of factors is incriminated in influencing these treatment outcomes. The factors include presence of high HCV RNA load, low treatment responses of genotypes 1 and 4, high rate replication (10^12^ virions/day) and its exceptionally high mutation rate producing a genetic variety [1]. Our result though does not have data on the influence of viral interaction, has shown that the presence of HCV decreases the efficiency of HAART when compare to those that were negative for HCV, hence goes well with the conclusions of Kedziora, *et al.* work [1]. The improvement of the immune status with CD4^+^ and CD8^+^ counts on its own improves survival of patients the minimum by slowing viral load and elimination of infected cells.

In the present study, the liver enzyme levels were much higher than the above limits of reference in co-infected than HIV mono-infected patients. In supporting the present study; DeSemone and his colleagues [13] found that increased liver enzyme level often associated with HCV co-infection. The mean GPT level of pre-ART co-infected group 4 patients during 1^st^ year was at least fivefold greater than that of under HAART co-infected group 2 patients. From 2^nd^ year and onwards, both group 2 and group 4 had comparatively increased levels of liver enzymes (Figure 3 and Table 4). In this study, more than 70% of the co-infected patients showed increased levels of GPT above the limits of reference which agreed with the finding of Lawson [14] in which only 30% of HCV patients have normal GPT levels.

Moreover, Sulkowski and his colleagues [6] found that HCV has been confirmed to be a risk factor associated with a 3 to 5-fold chance of developing elevated transaminases during HAART, compared to HIV patients without HCV which was compatible with the present study. However, the present work was incompatible with the study done by Gatti, *et al.*[8] that showed the synergetic mitochondrial damage by HCV and HAART (especially nucleoside analogs) were responsible for elevation of GPT in HAART groups. In addition, the higher enzyme level in the co-infected pre-ART group in this study indicates that the effect of HCV is more pronounced in pre-ART era than in the era of HAART. Furthermore, the HIV mono-infected group 1 and group 3 had normal amount enzyme levels throughout the follow up time. In supporting the normal enzyme levels of HIV mono-infected group 1 and group 3 in this study, Marina and Vincent [15] found that HAART do not associated with increased liver enzymes.

The present study has several strengths. Although viral load, HCV genotype and alcohol consumption status of patients are crucial in clinical medicine however, CD4^+^ count is the main test routinely done to follow immune recovery. The present study clearly showed the pattern of CD4^+^ changes considering different factors during the time of follow up. The information obtained in this study may promote our understanding of the impact of HCV infection on immunological parameters and liver enzyme levels among HIV/HCV co-infected individuals. Viral load, HCV genotype, alcohol consumption status and other clinical information of patients were not done in this study. Thus, with those limitations, we believe that this cohort may provide an accurate reflection of current clinical trends regarding this dual infection.

## Conclusion

The present study has shown that HCV infection has an impact on the recovery of CD4^+^ and CD8^+^ cells of on under HAART patients. The improvement in CD4^+^ cell count of under HAART HIV/HCV co-infected subjects were lower than the HIV mono-infected subjects and the mono-infected patients responded better to HAART than the co-infected patients. Moreover, HCV has a significant association with higher liver enzyme level than CD4^+^ in HIV/HCV co-infected patients. Because of the lower prevalence rate of HIV/HCV co-infection reported from previous few local studies in Ethiopia, the disease was given less attention and seems forgotten at various levels of health delivery institutions so that the significance of the problem has been underestimated. Therefore, it is advisable to make HCV-antibody screening for every HIV infected individual prior to initiation of HAART. This will in turn influence their clinical management as well as outcome.

## Competing interests

As authors, we declare that, we have no any competing interests.

## Authors’ contributions

The study was designed by ST. He also carried out data collection and laboratory works, performed data analysis and interpretation. ML revised the result critically and contributed to the final write up and finally, approved the manuscript.

## References

[B1] Kedziora P Figlerowicz M Formanowicz P Computational methods in diagnostics of chronic hepatitis C Bull Pol Ac Tech 2005 53 3 273 281

[B2] Addisu A Yayehyirad T Zufan S Techalew S Prevalence and risk factors of Hepatitis C among individuals presenting to HIV testing centers, Hawassa city, Southern Ethiopia BMC Research Notes, Short Report 2011 4 193 10.1186/1756-0500-4-193 PMC3138462 21676227

[B3] Mohsen A Easterbrook P Hepatitis C testing in HIV infected patients J Sex Trans Inf 2003 79 76 11 36 10.1136/sti.79.1.76 PMC1744592 12576627

[B4] Guo F Wei L Han Y Impact of Hepatitis C Virus Coinfection on HAART in HIV-Infected Individuals: Multicentric Observation Cohort J AIDS 2010 54 2 137 142 10.1097/QAI.0b013e3181cc5964 20431395

[B5] Chung R Management of HIV/HCV coinfection The PRN notebook 2004 9 1 14 19 http://www.prn.org

[B6] Sulkowski M Thomas D Chaisson R Moore R Drug-associated Liver Disease during HAART: impact of HCV Coinfection JAMA 2000 284 74 8010632283

[B7] Workenesh A Cutts F Nokes J Higher prevalence of anti-HCV antibodies among HIV-positive compared to HIV-negative inhabitants of Addis Ababa: results from a community based survey J Med Virol 2002 68 12 17 10.1002/jmv.10164 12210425

[B8] Gatti F Nasta P Matti A Treating Hepatitis C Virus in HIV Patients: Are Side Effects a Real Obstacle? AIDS Rev 2007 9 16 24 17474310

[B9] Larsen C Pialoux G Salmon D Prevalence of hepatitis C and hepatitis B infection in the HIV-infected population of France Eurosurveillance 2008 13 22 109 112 18761958

[B10] Brass V Moradpour D Blum H Molecular Virology of Hepatitis C Virus (HCV) Int J Med Sci 2006 3 29 34 16614739 10.7150/ijms.3.29 PMC1415840

[B11] Sugimoto K Stadanlick J Ikeda F Influence of Ethnicity in the Outcome of Hepatitis C Virus Infection and Cellular Immune Response J Hepat 2003 37 93 590 599 10.1053/jhep.2003.50103 12601357

[B12] Highleyman L Does HIV-HCV Coinfection Increase the Risk of Liver Disease Progression and Worsen Clinical Outcomes? HIV and Hepatitis.com 2008

[B13] DeSimone J Pomerantz R Babinchak T Inflammatory Reactions in HIV-1 Infected Persons after Initiation of Highly Active Antiretroviral Therapy Ann Intern Med 2000 133 447 454 10.7326/0003-4819-133-6-200009190-00013 10975963

[B14] Lawson A Hepatitis C, virus-infected patients with a persistently normal alanine aminotransferase: Do they exist and is this really a group with mild disease? J Viral Hepat 2010 17 1 51 58 19656289 10.1111/j.1365-2893.2009.01148.x

[B15] Marina N Vincent S How effective is HAART in HCV and HIV coinfection? Clin Infect Dis 2003 37 1678 1685 10.1086/379774 14689351

